# Temporal and spatial variations in the bacterial community composition in Lake Bosten, a large, brackish lake in China

**DOI:** 10.1038/s41598-019-57238-5

**Published:** 2020-01-15

**Authors:** Lei Zhang, Tingting Shen, Yu Cheng, Tingting Zhao, Li Li, Pengfei Qi

**Affiliations:** 1 School of Civil Engineering and Architecture, Chuzhou University, Chuzhou, 239000 China; 2 State Key Laboratory of Pharmaceutical Biotechnology, Nanjing University, Nanjing, China

**Keywords:** Microbial ecology, Limnology

## Abstract

The bacteria inhabiting brackish lake environments in arid or semi-arid regions have not been thoroughly identified. In this study, the 454 pyrosequencing method was used to study the sedimentary bacterial community composition (BCC) and diversity in Lake Bosten, which is located in the arid regions of northwestern China. A total of 210,233 high-quality sequence reads and 8,427 operational taxonomic units (OTUs) were successfully obtained from 20 selected sediment samples. The samples were quantitatively dominated by members of *Proteobacteria* (34.1% ± 11.0%), *Firmicutes* (21.8% ± 21.9%) and *Chloroflexi* (13.8% ± 5.2%), which accounted for more than 69% of the bacterial sequences. The results showed that (i) Lake Bosten had significant spatial heterogeneity, and TOC(total organic carbon), TN(total nitrogen) and TP(total phosphorus) were the most important contributors to bacterial diversity; (ii) there was lower taxonomic richness in Lake Bosten, which is located in an arid region, than in reference lakes in eutrophic floodplains and marine systems; and (iii) there was a low percentage of dominant species in the BCC and a high percentage of unidentified bacteria. Our data help to better describe the diversity and distribution of bacterial communities in contaminated brackish lakes in arid regions and how microbes respond to environmental changes in these stable inland waters in arid or semi-arid regions.

## Introduction

Heterotrophic bacteria are major constituents of pelagic aquatic ecosystems, where they play a prominent role in the breakdown of organic compounds and the remineralization of nutrients as well as in trophic coupling to eukaryote predators^1^. Recently, lakes, especially lakes in arid and semi-arid regions, have been described as early indicators of both regional and global environmental change. Microorganisms are some of the most important factors in the nutrient cycling and decomposition of organic matter in lake sediments^2^, and bacteria are thought to be a sensitive sentinel of those environmental changes^3^.

Sediments are some of the most diverse microbial habitats and play a key role in the biogeochemical cycles of organic matter decomposition and of major elements, including carbon, nitrogen and phosphorus. Sediment is now an major topic in the investigation of bacterial communities because analysis of the community in sediments can provide clues to understanding benthic ecosystem processes, particularly in saline and eutrophic aquatic environments. Sediment bacteria, due to their high population density (>108 cells/g), play an important ecological role in the biogeochemical cycle in lake ecosystems^4^. The biomass and community structure of sediment bacteria change due to changes in the physical, chemical and biological factors in water and sediment, so they can also be considered representative organisms indicating environmental changes^5^. The combination of abiotic factors and microorganisms in sediments has an important effect on nutrient balance in water.

Arid and semi-arid regions account for almost one-third of the world’s land area^6^, and lakes in these areas provide sparse but valuable water resources for fragile environments and humans. However, these water resources are also inevitably affected by human activities. Studies have shown that many water resources in arid and semi-arid regions have been contaminated by eutrophication or salinity^7–9^. Lake Bosten is the largest inland brackish lake in the arid regions of northwestern China, and it plays an important role in the surrounding terrestrial ecosystems. Surprisingly, the lake was a freshwater lake before the 1960s, but climate change and human activities over the past 50 years have developed it into a brackish lake with moderate nutrient levels^10,11^. Salinization and eutrophication continue to affect Lake Bosten. Therefore, Lake Bosten provides us with an exclusive opportunity to carefully study the potential response mechanisms of bacterial communities in the early stages of salinization and eutrophication. At the same time, due to anthropogenic agricultural and tourism activities, it also forms a nutritional gradient from malnutrition to eutrophication^12^, and this complex ecosystem is an ideal model for studying the response of bacterial communities to salinity and nutrient gradients^13^. This work is particularly important for the restoration of polluted dry lakes, as changes in microbial communities may be some of the more rapid agents of biological change.

Although a small number of studies have reported temporal changes in community composition in the sediments of the Lake Bosten, these studies have concentrated on a single sample from a single season^14^. There is little research on the variations over the four seasons across the whole lake. In this study, we used high-throughput sequencing technology and redundancy analysis to explore the association between spatial-temporal changes in Lake Bosten bacterial communities and environmental factors. The research objectives are to identify (1) Whether there is spatial-temporal heterogeneity in the bacterial communities of lake sediments under the dual effects of eutrophication and salinity and (2) Which environmental factors control the assembly of bacterial communities in the sediments of Lake Bosten.

## Materials and Methods

### Study area and sampling

Lake Bosten (86°19′~87°28′E, 41°46′~42°08′N, 1046 m above sea level) is located in the southern part of Bohu County, Xinjiang Province, in the lower reaches of the Kaidu River. The basin is located in the center of Eurasia. The combination of sunlight and heat, the annual average precipitation is 64.3 mm, and the annual average evaporation is 1,881.2 mm, resulting in an inland desert climate^15^. The lake covers an area of approximately 1,100 km^2^ ^16^ with an average water depth of 7 meters and a maximum depth of more than 16 meters^3^. The annual average temperature is about 7.9 °C, and the coldest temperature occurs in January (−12.7 °C). There is a four-month freeze season at Lake Bosten from December to March every year^15^. Mountain Tian in Xinjiang, which is the lowest point of the Basin Yan. Additionally, the lake covers a vast area of more than 1,100 km^2^ and has a maximum water depth of more than 16 m. Originating from the snow-covered Mountain Tian, the Kaidu River, with an annual average runoff volume of 34 × 10^8^ m^3^, supplies 85% of the water volume of Lake Bosten^17^. Moreover, as Lake Bosten is located in the centre of Eurasia, a good level of light and heat provide extremely favourable natural conditions for the development of the lake, allowing the basin to be rich in animal and plant resources. The Kaidu River is divided into two branches at the Baolang Sumu Water Diversion Project, which are respectively injected into two lakes of Lake Bosten. The Large Lake Area is 55 km from east to west and 20 km wide from north to south. At an altitude of 1,048.75 m, the water surface area is 1,002. 4 km^2^, volume is 88 × 10^8^ m^3^, average water depth is 7.38 m, the maximum depth is 16 m. The centre of lake area (HX) is located in the centre of the Large Lake Area and is highly disturbed by hydrodynamic forces, but is less affected by external influences. Huangshuigou area (HS) located in the northwest of the Large Lake Area and is enriched with nutrients (nitrogen and phosphorus) and salinity from agricultural irrigation and industrial wastewater discharged from the surrounding rivers. There are the highest nutrients and salinity in the area of Lake Bosten^4^. The Large Lake Area is regularly monitored by the Environmental Protection Bureau of Bayinguoleng Mongolian Autonomous Prefecture and has been continuously monitored for over ten years. Small Lake Area, also known as macrophyte-dominated area (SC), covers an area of about 300 km^2^, and is an important reed production base in China.

**Figure 1:**
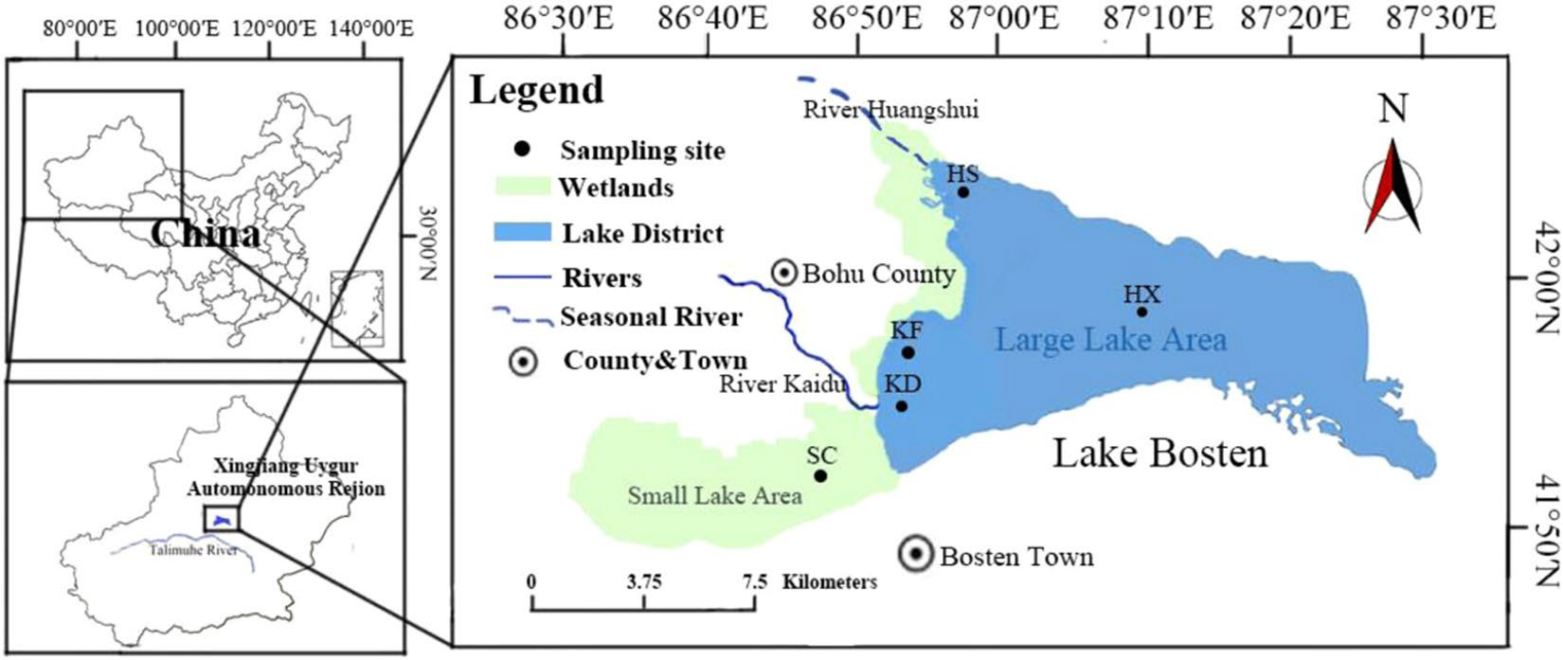
Map of the sampling sites in Lake Bosten, China.

From 8 May 2013 to 18 January 2014, 20 samples were collected from the centre of Lake area (HX, 87°08′00″E, 42°00′00″N), Huangshuigou area (HS, 86°50′30″E, 42°06′00″N), estuary of the Kaidu River (KD, 86°44′30″E, 41°53′40″N) and its nearby area (KF, 86°46′20″E, 41°57′00″N) and macrophyte-dominated area (SC, 86°38′5″E, 41°47′24″N). We avoided extreme weather when sampling and sampled when it was fine. Undisturbed duplicate sediments (0–5 cm deep) were collected seasonally using a Petersen grab sampler from 8 May 2013 to 18 January 2014 (Fig. 1). The sediment samples were transferred into sterile plastic containers, and immediately frozen at −80 °C for storage until analysis.

### Physicochemical analysis

The sediment samples were dried to constant weight using a freeze dryer (Alpha 1–2 LD, Martin Christ Instrument Co, Germany). Total nitrogen (TN), total phosphorus (TP) and total organic carbon (TOC) content in the sediments were determined according to standard methods (Jin and Tu 1990). The temperature, salinity, dissolved oxygen (DO), Salinity (TDS), and pH of the lowest water layer covering the sediment were measured using a multi-parameter water quality detector (YSI 6600V2, USA). After DAPI (4′, 6-diamidino-2-phenylindole) staining direct counting, the bacterial abundance in the deposited samples was determined by epifluorescence microscopy^12,15^. We used one-way ANOVA to test the correlation between environmental factors.

### DNA extraction, purification and PCR amplification

Total DNA was extracted from twenty 0.25 g freeze-dried pellets using the Soil Rapid DNA Rotation Kit (MP Biomedicals, Fountain Pkwy. Solon, OH, USA). Genomic DNA concentration and purity were determined by an ultraviolet spectrophotometer. According to the concentration test results, the integrity of the DNA sample was detected by 0.8% agarose gel electrophoresis. The test conditions were as follows: voltage 120 V, electrophoresis time was about 20 minutes.

The extracted DNA stock was diluted to 20 ng.μl^−1^ and used as a PCR template. The V1–V3 region of the bacterial 16S rRNA gene was amplified using PCR amplification of universal primers 8 F (5′-AGAGTTTGATCCTGGCTCAG-3′) and 533 R (5′-TTACCGCGGCTGCTGGCAC-3′)^18^. PCR amplification was carried out using a 50 μL reaction system under the following conditions: predenaturation at 94 °C for 5 minutes, and denaturation at 50 °C. Anneal at 94 °C for 30 seconds, 55 °C for 45 seconds, 72 °C for 1 minute, 27 cycles, 72 °C for 8 minutes, and stored at 4 °C, the experiment was repeated 3 times. The PCR product was purified and concentrated using E.Z.N.A. ^®^Cycle-Pure Kit. The PCR products from each sample were then mixed in a single tube in equimolar ratios.

### 454 pyrosequencing and data analysis

Amplicon pyrosequencing was performed on a Roche Genome Sequencer GS FLX Titanium platform using a 454/Roche A sequencing primer kit. Each read file produced by a pyrosequencing run is associated with one quality file, which contains the quality score for each base. After sequencing, the QIIME software package^19^ was used to exclude sequences with a length of less than 200 bases, an average mass fraction of less than 25, or no primers and barcode sequences. The rest of the sequences were trimmed and compared against the Bacterial Silva database (SILVA version 106; http://www.arb-silva.de/documentation/background/release-106/) via QIIME. Similar sequences were clustered into Operational Taxonomic Units (OTUs) using a minimum identity of 97% by UCLUST software^20^. Good’s coverage, abundance-based coverage estimator (ACE), Chao 1 richness estimator, Shannon and Simpson diversity indices were also calculated by QIIME pipeline.

## Results

### Physical and chemical factors

The environmental characteristics of the studied lake are presented in Fig. S1. The overlying water temperatures showed a characteristic annual cycle, with a higher temperature in the HS sample (26.9 °C, summer) and lower temperatures in the KF and SC samples (1.0 °C, winter). In the overlying water, the concentration of TDS increased from 278 mg L^−1^ in the KD sample (winter) to 2,238 mg L^−1^ in the HS sample (spring). The average concentrations of TN and TP in the seasonal sediment samples remained stable at approximately 1.74 (KD, winter) to 4.08 (SC, winter) mg·g^−1^ and 0.15 (KD, winter) to 0.52 (HS, winter) mg·g^−1^, respectively. The average concentration of TOC among the seasonal sediment samples ranged from 3.07 (HX, summer) to 12.4 (HS, summer) mg·g^−1^.

### Bacterial diversity

**Figure 2:**
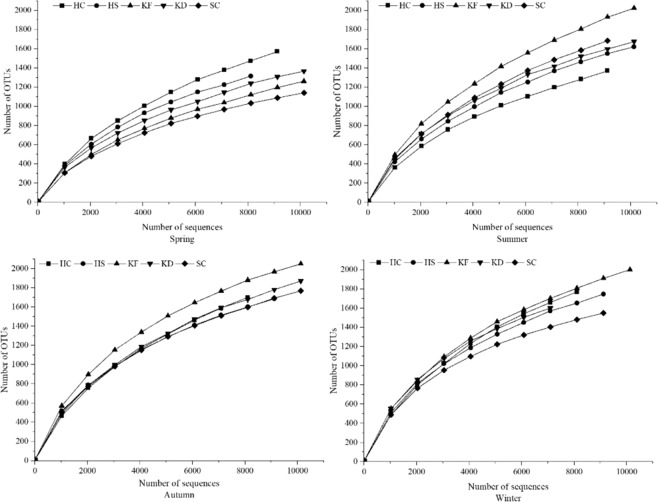
Rarefaction curves of OTUs clustered at 97% sequence identity across twenty samples in Lake Bosten.

The seasonal variation in the bacterial community structure in Lake Bosten was determined using 454 pyrosequencing technology. From all sediment samples, sequencing analysis yielded a total of 210,233 high-quality sequence reads with an average length of approximately 400 bp effective sequence, 8,427 OTUs, and 97% sequence identity threshold. The effective sequence length for each sediment sample ranged from 8,729 to 12,909, and the number of OTUs ranged from 1,319 to 2,209 (Table 1). The sparse curve with a cluster distance of 0.03 was still not saturated and increased (Fig. 2), indicating that new bacterial populations may continue to appear after 10,000 reads and sequencing. However, the values of the Shannon index (4.68–6.64) for all sediment samples in this study stabilized, suggesting that more sequencing may be required, but most of the bacterial diversity of the sample has been captured. In addition, good reports showed that these libraries represent the majority of bacterial 16S rRNA sequences present in each sediment sample, ranging from 0.902 to 0.951 (Table 1).

**Table 1:** Bacterial index of community diversity in Lake Bosten.

Site	Reads	OTUs	Chao 1	Ace	Simpson	Shannon	Coverage
HX (Spring)	9687	1613	2724	3285	0.05	5.26	0.919
HS (Spring)	8917	1371	2067	2156	0.04	5.24	0.931
KF (Spring)	11350	1319	2121	2572	0.06	4.68	0.947
KD (Spring)	12600	1521	2470	3215	0.02	5.50	0.945
SC (Spring)	10483	1158	1852	1804	0.03	4.85	0.951
HX (Summer)	9895	1416	2238	2305	0.04	5.13	0.933
HS (Summer)	11228	1706	2737	3383	0.03	5.49	0.929
KF (Summer)	10381	2048	3323	4198	0.01	6.22	0.905
KD (Summer)	12909	1838	2546	2647	0.01	6.14	0.945
SC (Summer)	9545	1714	2749	2837	0.03	5.65	0.915
HX (Autumn)	8809	1770	2818	3656	0.03	5.73	0.902
HS (Autumn)	9210	1692	2529	2586	0.00	6.42	0.923
KF (Autumn)	12520	2209	2949	2960	0.00	6.64	0.938
KD (Autumn)	12383	2038	3040	3114	0.01	6.28	0.930
SC (Autumn)	12416	1911	2744	2762	0.01	6.35	0.940
HX (Winter)	8729	1836	2790	2946	0.03	5.94	0.903
HS (Winter)	9128	1746	2545	2684	0.00	6.46	0.919
KF (Winter)	12829	2193	2931	3055	0.00	6.58	0.937
KD (Winter)	7860	1663	2245	2305	0.00	6.51	0.920
SC (Winter)	9354	1562	2083	2113	0.01	6.23	0.941

### Bacterial community composition

**Figure 3:**
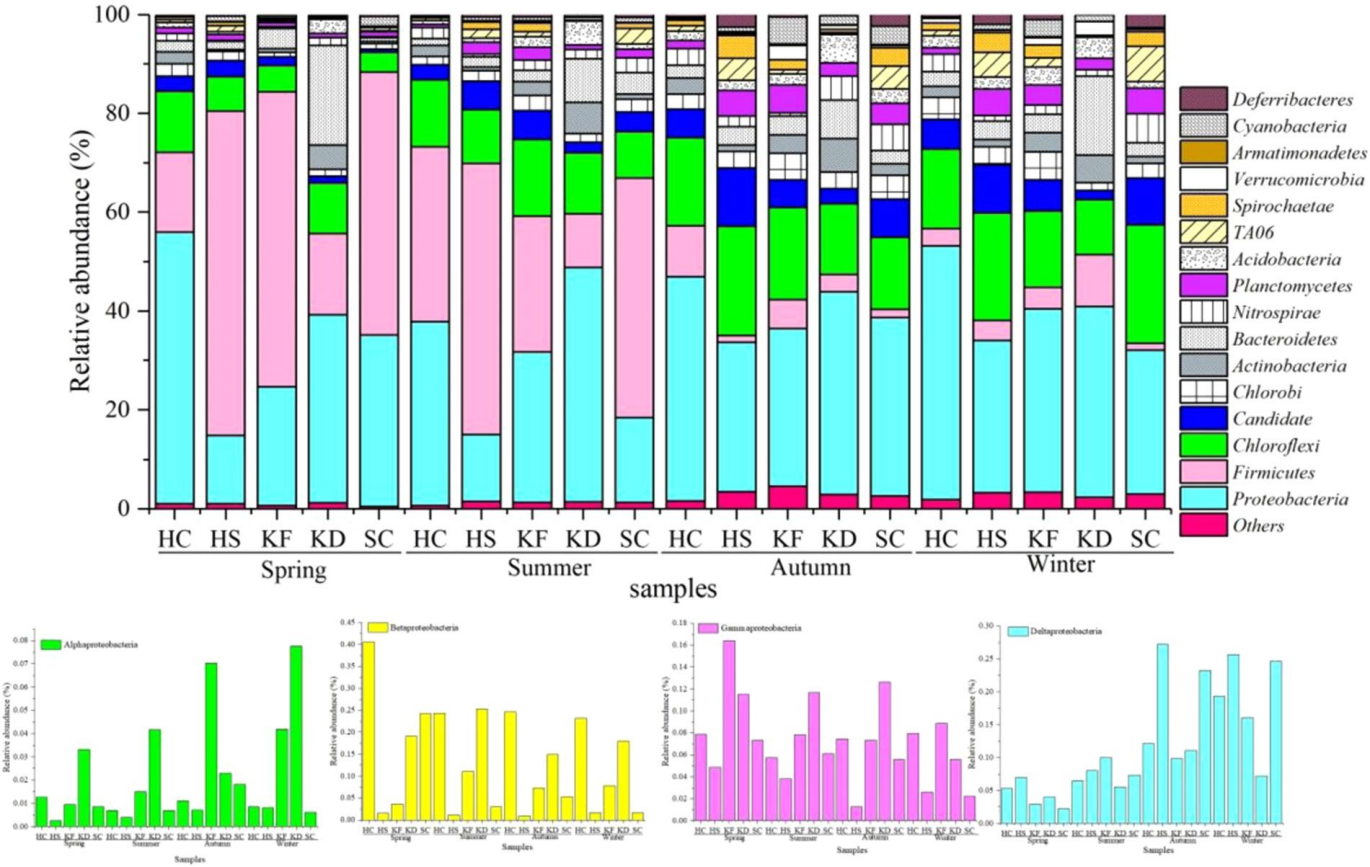
Relative abundances of top 10 phyla or sub-phyla in each sample (a total of 15 phyla or classes for all twenty sedimentary samples) from Lake Bosten.

In the filtered high-quality sequences, the dominant bacterial phylum or sub-phylum in each sediment sample was identified by the SILVA database using the QIIME algorithm (the top 10 most abundant phyla or sub-phyla in each sample). The most abundant bacterial phyla were *Proteobacteria* (34.1% ± 11.0%), *Firmicutes* (21.8% ± 21.9%) and *Chloroflexi* (13.8% ± 5.2%), representing more than 69.0% of the bacterial sequences (Fig. 3). In addition, a large number of bacteria were detected at lower numbers (<4% relative abundance per phyla) in the sediment samples, such as *Planctomycetes*, *TA06*, *Candidate division WS3*, *Bacteroidetes, Spirochaetae*, *Chlorobi*, *OD1*, *OP8* and *Deferribacteres* (Fig. 3). *Firmicutes* (10.9%–65.7%) dominated in the spring and summer samples. In contrast, the autumn and winter samples were mainly dominated by *Deltaproteobacteria* (0.19%–27.2%) and *Chloroflexi* (0.18%–22.1%) (Fig. 3).

**Figure 4:**
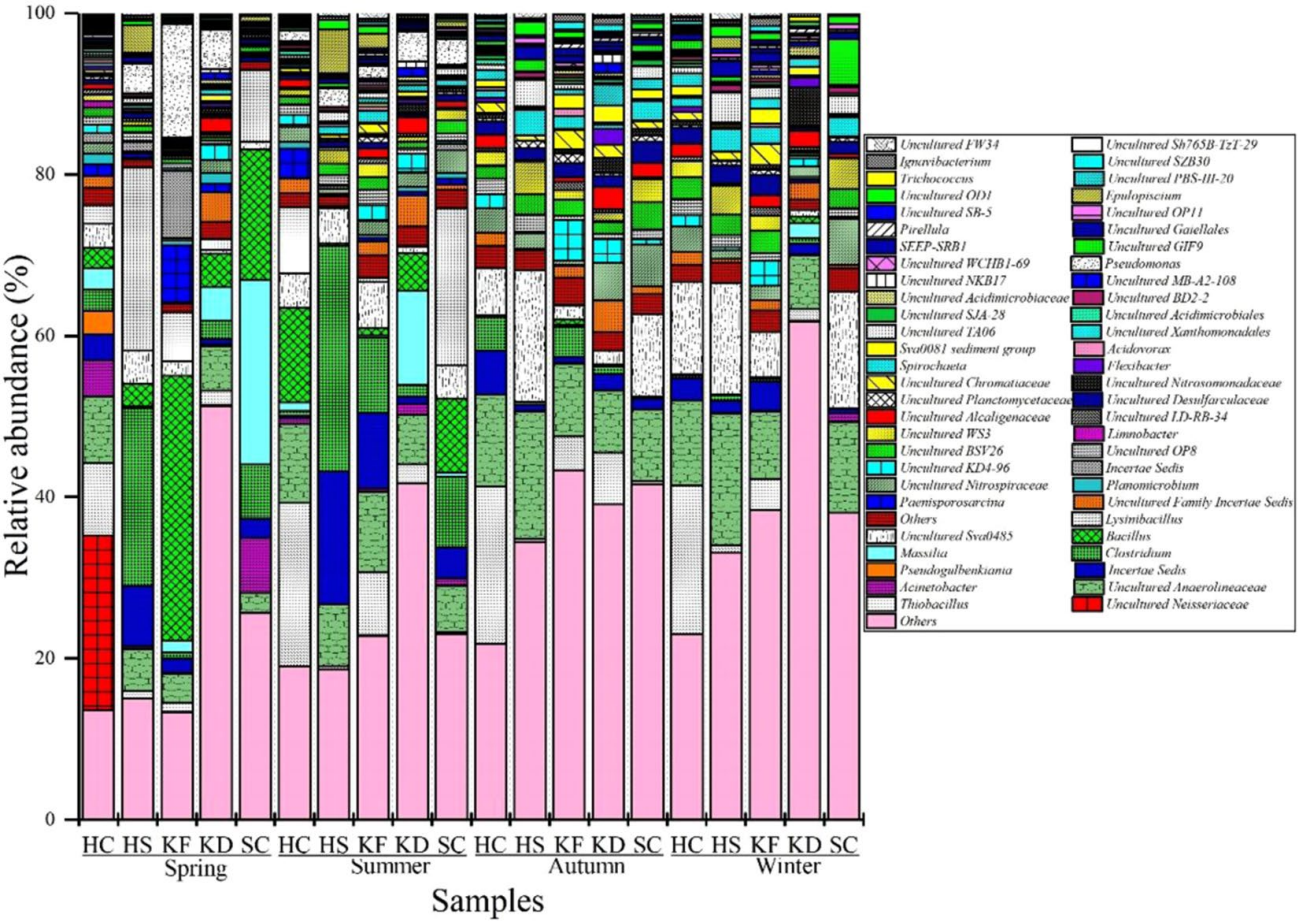
Comparison of percentage of the sequences affiliated with the frequently identified genera to the total number of sequences from twenty sedimentary samples in Lake Bosten.

Figure 4 shows the 56 dominant genera in the sediment samples. At the genus level, the differences between the sediment samples are more pronounced. Microorganisms from the genera Uncultured *Neisseriaceae* (21.62%) and *Lysinibacillus* (22.63%) predominated in the BS1 and BS2 samples but were less abundant in the other samples. The genus *Clostridium* (27.94%) and unclassified *Peptostreptococcaceae* (16.47%) had higher proportions in the HS (summer) sample. Unclassified *Anaerolineaceae* (2.42%–16.38%) and *Sva0485* (0.77%–16.33%) were more abundant in SC (spring) and KD (summer) samples. Therefore, the main bacterial populations in the twenty sediment samples are slightly different.

### Redundancy analysis of sediment bacteria and environmental factors

**Figure 5:**
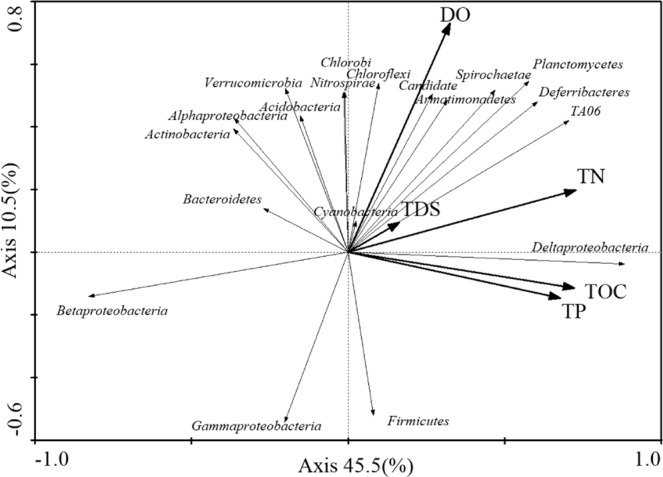
Analysis (RDA) of bacteria community with water quality. Correlation between the relative abundance of dominant bacteria and main sediment parameters (**a**) in phylum level; (**b**) in class level).

Microorganisms are highly sensitive to environmental changes, and the relationship between different environmental factors and the microflora structure can be determined according to the size of the angle between bacterial and environmental factors and the length of the connection in a redundancy analysis (Fig. 5). *Betaproteobacteria*, *Firmicutes*, and *Gammaproteobacteria* were negatively correlated with TDS and DO, whereas *Chloroflexi* and *Bacteroidetes* were positively correlated with TDS and DO. *Deltaproteobacteria* was positively correlated with each indicator. In the RDA analysis diagram, combining the sediments showed that the levels of the physical and chemical indicators phylum flora structure, TN, TOC and TP were the main factors that influenced the growth of bacteria, but other environmental factors also had certain effects.

## Discussion

The five sampling points selected from the Large Lake area and Small Lake area in Lake Bosten are representative areas for studying the sediment bacterial community structure of Lake Bosten. Based on the results of the physical and chemical analyses, the physical and chemical indicators of the 20 samples showed certain similarities and differences due to factors such as time and geographical location. There was no significant change in the pH value among the 20 samples, but the other indicators had significant differences (Fig. S1). Most obviously, the values of TDS at the Huangshuigou and central lake areas were significantly higher than those at the other sampling points (P < 0.05). According to previous research, agricultural production around Lake Bosten discharges a large amount of farmland drainage with high salinity that includes chloride ions, sulfate ions, ammonia nitrogen and so on. This discharge was the main reason for the increased salt content in Lake Bosten, as 71% and 29% of the lake water flows into the Large Lake Area and Small Lake Area, respectively^21^. In addition, the TDS of the summer and autumn samples from the estuary of Lake Bosten and nearby areas was higher than the TDS in other seasons. Weberscannell *et al*.^22^ noted that changes in the concentrations of TDS in natural waters are often attributed to the discharge of industrial effluent, increased precipitation, or saltwater intrusion. In the summer and autumn, the Kaidu River glaciers melted and created abundant snow and ice water resources, which caused the water volume to increase rapidly^23^, leading to a significant increase in TDS content. However, the specific causes of this difference in TDS could be complex, and other potential reasons for this difference still deserve further investigation. Interestingly, the contents of TD, TP and TOC in the Huangshuigou and macrophyte-dominated area were higher in winter than in summer (Fig. S1), which may be because the continuous low temperature in winter inhibits microbial activity and reduces the effect of microorganisms on the removal of nitrogen and phosphorus^24^. On the other hand, TN and TP concentrations were reduced due to dilution and biological blocking^25^. In addition, a large number of aquatic plants in the aquatic grass area can enrich N and P and desalinize water by adsorbing, decomposing, oxidizing and precipitating the water, salt, and N and P^26^. This also explained why the value of pollution indicators in the macrophyte-dominated area was higher than that in the Huangshuigou area.

A large number of studies have shown that temperature is an important factor affecting the structure of bacterial communities^27^. High temperatures can increase the metabolic rate, thus causing the cycle of organic matter to increase in speed; a drop in temperature will lower the growth rate of bacteria and lower productivity^27^. This trend has also been confirmed in the laboratory^28^. Therefore, the bacterial community composition of the samples in autumn and winter was more complicated than that in the samples in spring and summer (Fig. 3). This may be due to the relative abundance of the dominant bacteria *Firmicutes* in spring and summer decreasing greatly under the influence of temperature. Temperature changes improve the competitive advantage of other bacteria and increase the relative abundance of other bacteria, leading to a higher diversity of bacteria in autumn and winter. The competitive advantage between microorganisms changes under conditions such as temperature changes^29,30^, and some studies on bacteria also illustrate this point. In aquatic ecosystems, in addition to temperature, the most relevant factor to bacterial growth and reproduction is inorganic salt nutrition (such as TN, TP) and algae biomass^31^. Shiah′s study also suggests that in the Chesapeake at temperatures below 20 °C, temperature, more than nutrient levels, functions as the main control factor of planktonic bacteria. At temperatures above 20 °C, the nutrient control became dominant^5^.

In the whole bacterial community, the three most abundant phyla were *Firmicutes* (21.8% ± 21.9%), *Proteobacteria* (*Deltaproteobacteria* and *Gammaproteobacteria*) (34.1% ± 11.0%) and *Chloroflexi* (13.8% ± 5.2%), accounting for more than 69.0% of the bacterial community. This community structure is largely in accordance with those found in other sediments and soils worldwide^32,33^ (Fig. 3).

*Firmicutes* (10.9%–65.7%) dominated sediment samples in spring and summer but was present at less than 1% abundance in autumn and winter. The RDA suggested that the members of *Firmicutes* were not particularly dependent on the nutrients in the seasonal samples (Fig. 5). This finding was consistent with that from a previous study in Lake Dongping, a shallow lake^34^. Under anaerobic conditions, nitrate can be used to achieve denitrification^35^. DO in spring and summer was lower than that in autumn and winter, suggesting that the increase in DO would restrain the growth of *Firmicutes*. Related studies have shown that DO controls the *Firmicutes* community structure more than other main factors, which is consistent with the results of RDA analysis (Fig. 5). The genus *Clostridium* are specialized anaerobic bacteria that mainly exist in anaerobic environments and have strong degradation ability and high metabolic activity^36^. The genus *Bacillus* was the primary component of *Firmicutes*, and the group was abundant in the spring and summer samples (Fig. 4). A previous study observed significant variations in the composition of the genus *Bacillus* in different estuary sediments from Lake Taihu^37^, and their presence caused the production of high amounts of ammonia, nitrite and other hazardous substances^38^. The genus *Lysinibacillus*, which includes facultative bacteria, can reduce macromolecular organic matter to low molecular weight acids, dissolve nutrients in the soil sediment^39^, degrade crude compounds^40^, fix nitrogen from the air^41^ and prevent plant diseases and insect pests^39^, and therefore has certain environmental benefits.

*Proteobacteria* (13.5%–55.0%) were the dominant bacteria in all sediment samples. However, in samples from different seasons, the relative abundances of different kinds of *Proteobacteria* were very different (Fig. 3). In this study, *Deltaproteobacteria* were the main group of *Proteobacteria*, followed by *Gammaproteobacteria* and *Betaproteobacteria* (Fig. 3). In the sediment samples from autumn and winter, *Deltaproteobacteria* dominated. In freshwater sediments, *Deltaproteobacteria* were also abundant^6,42^. *Deltaproteobacteria* was mainly represented by unclassified *Desulfarculaceae* in the present study (Fig. 4). As sulfate-reducing bacteria, the family *Desulfarculaceae* has been detected as being dominant in the salt marsh sediments of the North Atlantic and on the East Coast of the US. These bacteria are important participants in the sulfur biogeochemical cycle^43,44^ and can degrade organic matter. The resultant compensatory nutrition and energy sources play an important ecological role, especially in the anaerobic degradation of organic matter and the conversion process^45,46^. In the past 20 years, the chemicals in Lake Bosten have come to include high levels of sodium sulfate^47^; a high concentration of SO_4_^2−^ and TDS provide good nutrient conditions, thereby promoting the growth of bacteria. Therefore, its dominant position in the sample may be related to nutrients in the lake.

As a second group of *Proteobacteria*^48,49^, *Gammaproteobacteria* play an important role in the degradation and absorption of organic compounds^50^, ammonia^51^ and sulfide^52,53^ (Fig. 3). The relative abundance of *Gammaproteobacteria* (1.27–16.40%) in the estuary and its vicinity was significantly higher than that in the other samples. Several previous studies have revealed that *Gammaproteobacteria* usually exist in sediments in areas contaminated with agricultural pollution or organic matter^15,54^. The agricultural activities around Lake Bosten have increased the amounts of organic pollutants in the estuary^55^, and the organic content of the sediments has increased^56^. Therefore, it was not surprising that *Gammaproteobacteria* were abundant in the estuarine sediments of Lake Bosten, and a high relative abundance of *Gammaproteobacteria* has also been observed in other aquatic systems^57,58^. Additionally, the genus *Pseudomonas* was found to be representative of *Gammaproteobacteria*. The group appeared as the dominant genus in the estuarine sediment samples, especially in the spring sample (Fig. 4). A high relative abundance of the genus *Pseudomonas* has been observed in the estuarine sediments of Lake Poyang^59^. In addition, the genus *Acinetobacter* was a minor proportion of the *Gammaproteobacteria* (Fig. 4). A previous study indicated that the genus *Acinetobacter* is commonly detected as phosphorus-accumulating microorganisms in sediments^60^ and that the presence of the genus *Acinetobacter* might explain the phosphorus-accumulating microorganism concentration in the phosphorus-containing sediment samples from summer (Fig. S1)^59^.

In this study, *Betaproteobacteria* appeared as the predominant phylum in all sediment samples (Fig. 3). This finding was consistent with that of a previous study in which *Betaproteobacteria* was found to be the predominant group in the upper sediments of Lake Taihu^61^. *Betaproteobacteria* (0.87–40.56%) are a dominant bacterial group widely distributed in freshwater lakes around the world. They are present in surface waters and are one of the most studied groups to date^62^, but they are rarely found on the open seas or in salt lakes^2,63^. The presence and success of *Betaproteobacteria* in freshwater lakes are related to their ability to respond quickly to changes in bioavailable nutrient content, e.g., they have a rapid response to dissolved organic carbon^64^. However, the results of the RDA analysis in this study showed that nutrients such as TOC, TP and TN did not exhibit positive correlations with the relative abundance of *Betaproteobacteria* (Fig. 5). Interestingly, the most abundant OTU in *Betaproteobacteria* was the genus *Thiobacillus*. The genus *Thiobacillus* is a dominant genus in habitats polluted with mine wastewater^65^, and they are very abundant in all four seasons. *Thiobacillus* was the dominant genus in the HS samples. This indicates that there may be substantial mineral pollution in the HS area.

*Chloroflexi* (3.94–22.13%) was evenly distributed among the various sediment samples. A previous study indicated that members of *Chloroflexi* are active not only in deep subsurface sediments on the ocean floor^66^ but also in various wastewater treatment systems^67^. In this study, the RDA showed a positive correlation between the concentration of TOC and the relative abundance of *Chloroflexi* (Fig. 5). This finding was consistent with that of a previous study that reported on riparian sedimentary ecosystems^34^. *Chloroflexi* can biodegrade organic pollutants^68^, giving it a better growth advantage in eutrophic environments^69^. In addition, *Chloroflexi* efficiently degrades chlorides. Zanaroli *et al*.^70^ found that the genus does not produce O_2_ by photosynthesis and cannot fix nitrogen but has a dechlorination function. Unclassified *Anaerolineaceae* was dominant in *Chloroflexi*, and the group dominated in all seasonal sediment samples (Fig. 4). The family *Anaerolineaceae* has previously been found to be dominant in the estuarine sediments of Lake Taihu^69^, and members of this family are often involved in the anaerobic degradation process of alkanes^71,72^, as well as in the biodegradation process of organic pollutants^59,73^.

Bacteroidetes were dominant in samples from all seasons (Fig. 3). A previous study revealed that Bacteroidetes has the capacity to decompose complex molecules in freshwater sediments^74^. Furthermore, our results indicated that the genus Flavobacterium and unclassified vadinHA17 were the two primary components of Bacteroidetes, of which the genus Flavobacterium was the most abundant OTU. A similar study revealed that the genus Flavobacterium functions in heterotrophic nitrification and metabolizes refractory organic compounds^75^. Therefore, the enrichment of the genus Flavobacterium may indicate widespread organic pollution in Lake Bosten.

It is noteworthy that there was a substantial percentage of unclassified bacterial groups detected in these seasonal samples (Fig. 4). In addition to those mentioned above, there were still several dominant unclassified bacteria found in the samples, such as unclassified *Caldilineaceae*, unclassified *KD4–96*, and some incertae cedis bacteria (Fig. 4). Numerous studies have observed the abundance of unclassified bacteria in various lake systems^76,77^, especially in sulfur-rich and anoxic environments^18,78^. The existence of abundant unclassified species in the estuarine sediments of Lake Bosten indicates that new bacterial communities inhabit Lake Bosten and that the bacterial diversity, taxonomy and phylogenetics of the location deserve further attention.

## Conclusion

In this study, 454 pyrosequencing technology was used to study the bacterial community structure of the sediments in Lake Bosten. We found that the bacterial community of Lake Bosten sediments is spatially specific. For example, the genus *Thiobacillus*, which is mainly found in mine wastewater and had a relatively high abundance in other areas, was found in the HS area; enrichment in the HS area may indicate that the area is rich in minerals. In addition, there are obvious seasonal changes in the bacterial community of sediments. For example, the relative abundance of the Firmicutes in the SC samples in the spring sampling was 53.3%, but only 0.14% in winter. The discharge of agricultural sewage caused the TN and TP of the HS area to be higher than those of other samples, and the adsorption and decomposition of aquatic plants led to the enrichment of pollutants in the SC area. Furthermore, temperature and nutrient status are key factors driving the bacterial community structure in sediments. We have discovered nitrifying bacteria with a large amount of degradable pollutants in Bosten Lake, and our research provides a reference for preventing and remediating lake pollution.

## Supplementary information

Supplementary Information: Environmental variables from twenty sediments in Lake Bosten.

## References

[1] Azam F (1998). Microbial control of oceanic carbon flux: the plot thickens. Science.

[2] Tamaki H, Sekiguchi Y, Hanada S (2005). Comparative analysis of bacterial diversity in freshwater sediment of a shallow eutrophic lake by molecular and improved cultivation-based techniques. Appl. Environ. Microb..

[3] Newton RJ (2011). A guide to the natural history of freshwater lake bacteria. Microbiol. Mol. Biol. R..

[4] Nealson KH (1997). Sediment bacteria: who’s there, what are they doing, and what’s new?. Annu. Rev. Earth Pl. Sc..

[5] Zhang R, Thiyagarajan V, Qian PY (2008). Evaluation of terminal-restriction fragment length polymorphism analysis in contrasting marine environments. FEMS Microbiol. Ecol..

[6] Wang, H. Optimized estimation and its uncertainties of gross primary production over oasis-desert ecosystems in an arid region of China. *Agu. Fall. Meeting*. (2017).

[7] Huo S (2015). Establishing water quality reference conditions for nutrients, chlorophyll a and Secchi depth for 7 typical lakes in arid and semiarid ecoregion, China. Environ. Earth Sci..

[8] Cardona A (2004). Salinization in coastal aquifers of arid zones: an example from Santo Domingo, Baja California Sur, Mexico[J]. Environ. Geol..

[9] Liu H (2013). Disappearing Lakes in Semiarid Northern China: Drivers and Environmental Impact. Environ. Sci. & Technol..

[10] Sai B (2011). Response of planktonic bacterial abundance to eutrophication and salinization in Lake Bosten, Xinjiang. J. Lake Sci..

[11] Tang X (2012). Influence of salinity on bacterial community composition in Lake Bosten, a large oligosaline lake in the arid northwestern China. Appl. Env. Microb..

[12] Xie G (2011). Spatio-temporal heterogeneity of water quality and succession patterns in Lake Bosten during the past 50 years. J. Lake Sci..

[13] Tang X (2012). Influence of salinity on the bacterial community composition in Lake Bosten, a large oligosaline lake in arid northwestern China. Appl. Environ. Microbiol..

[14] Shi Z (2012). Characterization of the underwater light field and the affecting factors in Bosten Lake in summer. Acta Scientiae Circumstantiae.

[15] Zhang L (2016). Pyrosequencing analysis of bacterial communities in Lake Bosten, a large brackish inland lake in the arid northwest of China. Can. J. Microbiol..

[16] Mischke S, Wünnemann B (2006). The Holocene salinity history of Bosten Lake (Xinjiang, China) inferred from ostracod species assemblages and shell chemistry: possible palaeoclimatic implications. Quatern Int..

[17] Xie G (2011). Spatio-temporal heterogeneity of water quality and succession patterns in Lake Bosten during the past 50 years. Lake Sci..

[18] Bai Y (2012). Bacterial communities in the sediments of Dianchi Lake, a partitioned eutrophic waterbody in China. PLoS one.

[19] Caporaso JG (2010). QIIME allows analysis of high-throughput community sequencing data. Nat. Methods.

[20] Edgar RC (2010). Search and clustering orders of magnitude faster than BLAST. Bioinformatics.

[21] Sai B, Chen MP, Feng L (2012). Agricultural non-point source pollution of Bosten Lake basin (in Chinese). Water Resour. Prot..

[22] Weberscannell PK, Duffy LK (2007). Effects of Total Dissolved Solids on Aquatic Organisms: A Review of Literature and Recommendation for Salmonid Species.. Ameri. J. Env. Sci..

[23] Wu J, Ma L, Zeng H (2013). Analysis on water quality and water quantity of Lake Bosten in xinjiang and its evolution characteristics. J. Geogr. sci..

[24] Liu, T. T. The seasonally change and output of carbon, nitrogen and phosphorus in the waterbody of Jialing River (In Chinese). Chongqing, Southwest Universe (2009).

[25] Guoming Z, Yang G, Zhaojun L (2008). Study on non⁃ point resource dynamic transport of Lanhe watershed located in Fenhe River s upstream. J. Soil. Water Conserv..

[26] Chen MX (2014). N, P and salt contents in hydrophytes in the Bostern Lake, Xinjiang (in Chinese). Arid. Zone Res..

[27] White PA (1991). The effect of temperature and algal biomass on bacterial production and specific growth rate in freshwater and marine habitats. Microb. Ecol..

[28] Kirchman DL, Rich JH, Barber RT (1995). Biomass and biomass production of heterotrophic bacteria along 140 W in the equatorial Pacific: effect of temperature on the microbial loop. Deep-Sea Res. Pt II.

[29] Eduok S (2017). Aged-engineered nanoparticles effect on sludge anaerobic digestion performance and associated microbial communities. Sci. Total. Environ..

[30] He S (2018). Effects of temperature on anammox performance and community structure. Bioresour. Technol..

[31] Smith R (2002). Limnology—Inland water ecosystems. J. N. Am. Benthol. Soc..

[32] Shao K (2011). Comparing sediment bacterial communities in the macrophyte-dominated and algae-dominated areas of eutrophic Lake Taihu, China. Can. J. Microbiol..

[33] Zaitseva SV (2014). Microbial community of the bottom sediments of the brackish Lake Beloe (Transbaikal region). Microbiology.

[34] Song H (2012). Bacterial communities in sediments of the shallow Lake Dongping in China. J. appl. microbiol..

[35] Luo J (2013). Microbial community structures in a closed raw water distribution system biofilm as revealed by 454-pyrosequencing analysis and the effect of microbial biofilm communities on raw water quality. Bioresour. Technol..

[36] Brown J (1984). Serum bone Gla-protein: A specific marker for bone formation in postmenopausal osteoporosis. Lancet.

[37] Klepacceraj V (2004). High overall diversity and dominance of microdiverse relationships in salt marsh sulphate-reducing bacteria. Env. Microbiol..

[38] Angermeyer A, Crosby SC, Huber JA (2016). Decoupled distance–decay patterns between dsr A and 16S r RNA genes among salt marsh sulfate‐reducing bacteria. Env. microbiol..

[39] Ferreira J, Matthee F, Thomas A (1991). Biological control of eutypa lata on grapevine by an antagonistic strain of Bacillus subtilis. Phytopathology.

[40] Yang, S. Q., Li, S. Y. & Li, F. Y. Selection of microflora and dominant bacteria degrading petroleum hydrocarbon in frozen soil in Shenfuzhi irrigation area. *J. Meteorol. Environ.*, 54–56 (2006).

[41] Xu, Q. F., Huang, X. L. & Chen, T. W. Classification and determination of nitrogen fixation activity of eight strains of Bacillus strains. *Microbiol. China*, 253–258 (1998).

[42] Spring S (2000). Identification and characterization of ecologically significant prokaryotes in the sediment of freshwater lakes: molecular and cultivation studies. FEMS Microbiol. Rev..

[43] Porter KG, Feig YS (1980). The use of DAPI for identifying and counting aquatic microflora. Limnol. Oceanogr..

[44] Shibata A (2007). Anaerobic biodegradation of 4-alkylphenols in a paddy soil microcosm supplemented with nitrate. Chemosphere.

[45] Bowman JP, McCuaig RD (2003). Biodiversity, community structural shifts, and biogeography of prokaryotes within Antarctic continental shelf sediment. Appl. Env. Microb..

[46] Soltwedel T, Vopel K (2001). Bacterial abundance and biomass in response to organism-generated habitat heterogeneity in deep-sea sediments. Mar. Ecol- Prog. Ser..

[47] Li WH, Yuan L (2002). Water and salt changes and their influencing factors in Lake Bosten, Xinjiang (In Chinese). J. Lake sci..

[48] Taoka A (2014). Characterization of uncultured giant rod-shaped magnetotactic Gammaproteobacteria from a freshwater pond in Kanazawa, Japan. Microbiology.

[49] Williams KP (2010). Phylogeny of gammaproteobacteria. J. Bacteriol..

[50] Disayathanoowat T (2012). Isolation and characterization of bacteria from the midgut of the Asian honey bee (Apis cerana indica). J. Apic. Res..

[51] Padhi SK (2013). Characterisation of heterotrophic nitrifying and aerobic denitrifying Klebsiella pneumoniae CF-S9 strain for bioremediation of wastewater. Int. Biodeter Biodegr.

[52] Cai J, Jiang J, Zheng P (2010). Isolation and identification of bacteria responsible for simultaneous anaerobic ammonium and sulfate removal. Sci. China Chem..

[53] Marshall KT, Morris RM (2013). Isolation of an aerobic sulfur oxidizer from the SUP05/Arctic96BD-19 clade. ISME J..

[54] Beardsley C (2011). Quantitative role of shrimp fecal bacteria in organic matter fluxes in a recirculating shrimp aquaculture system. FEMS Microbiol. Ecol..

[55] Zheng, B. *et al*. Records of organic carbon and nitrogen stable isotopes of lake environment changes in the past 200 years in Lake Bosten. 2012 **32**, 165–171 (2012).

[56] Huang W (2017). Characterization of sediment bacterial communities in plain lakes with different trophic statuses. MicrobiologyOpen.

[57] DeLong EF (2005). Microbial community genomics in the ocean. Nat. Rev. Microbiol..

[58] Friedline C (2012). Bacterial assemblages of the eastern Atlantic Ocean reveal both vertical and latitudinal biogeographic signatures. Biogeosciences.

[59] Sheng P (2016). Bacterial diversity and distribution in seven different estuarine sediments of Poyang Lake, China. Env. Earth Sci..

[60] Martiny JBH (2006). Microbial biogeography: putting microorganisms on the map. Nat. Rev. Microbiol..

[61] Liu L (2010). Vertical Structure of Bacterial and Archaeal Communities within the Sediment of a Eutrophic Lake as Revealed by Culture-Independent Methods. J. Freshw. Ecol..

[62] Tang X (2010). Dynamics of organic‐aggregate‐associated bacterial communities and related environmental factors in Lake Taihu, a large eutrophic shallow lake in China. Limnol. Oceanogr..

[63] Barberán A, Casamayor EO (2010). Global phylogenetic community structure and β-diversity patterns in surface bacterioplankton metacommunities. Aquat. Microb. Ecol..

[64] Newton RJ (2006). Microbial community dynamics in a humic lake: differential persistence of common freshwater phylotypes. Env. Microbiol..

[65] Fortin D, Davis B, Beveridge T (1996). Role of Thiobacillus and sulfate‐reducing bacteria in iron biocycling in oxic and acidic mine tailings. FEMS Microbiol. Ecol..

[66] Huber JA (2006). Microbial life in ridge flank crustal fluids. Env. Microbiol..

[67] Björnsson L (2002). Filamentous Chloroflexi (green non-sulfur bacteria) are abundant in wastewater treatment processes with biological nutrient removal. Microb.

[68] Xue YQ, Liu F, Jiang XD (2008). Diversity of winter sediment bacterial communities in different lake areas of Lake Taihu (In Chinese). China Env. Sci..

[69] Wu H (2017). Sediment bacterial communities in a eutrophic lake influenced by multiple inflow-rivers. Env. Sci. Pollut. R..

[70] Zanaroli G (2012). A Chloroflexi bacterium dechlorinates polychlorinated biphenyls in marine sediments under *in situ*-like biogeochemical conditions. J. Hazard. Mater..

[71] Chen R (2016). Evolution of the microbial community of the biofilm in a methane-based membrane biofilm reactor reducing multiple electron acceptors. Env. Sci. Pollut. R..

[72] Liang B (2015). Anaerolineaceae and Methanosaeta turned to be the dominant microorganisms in alkanes-dependent methanogenic culture after long-term of incubation. AMB. Express.

[73] Xiong, W. *et al*. Sources of organic matter affect depth-related microbial community composition in sediments of Lake Erhai, Southwest China. *J. limnol.* **74** (2014).

[74] Kirchman DL (2002). The ecology of Cytophaga–Flavobacteria in aquatic environments. FEMS Microbiology Ecol..

[75] Zhao B (2010). Heterotrophic nitrogen removal by a newly isolated Acinetobacter calcoaceticus HNR. Bioresour. Technol..

[76] Peter H (2018). Changes in bacterioplankton community structure during early lake ontogeny resulting from the retreat of the Greenland Ice Sheet. ISME J..

[77] Zhang H (2018). Water Bacterial and Fungal Community Compositions Associated with Urban Lakes, Xi’an, China. Inter. J. Env. Res. Pu. Heal..

[78] Klepacceraj V (2012). Microbial diversity under extreme euxinia: Mahoney Lake, Canada. Geobiology.

