# Supplementary material for: Temporal and spatial variations in the bacterial community composition in Lake Bosten, a large, brackish lake in China
Source: Sci Rep. 2020 Jan 15;10:304. doi: 10.1038/s41598-019-57238-5 (PMC6962391; doi:10.1038/s41598-019-57238-5)
Supplement: Supplementary file 1 — Supplementary Information: Environmental variables from twenty sediments in Lake Bosten. [file 41598_2019_57238_MOESM1_ESM.docx]

**Temporal and spatial variations in the bacterial community composition in Lake Bosten, a large, brackish lake in China**

Lei Zhang^1,^ *, Tingting Shen^1^, Yu Cheng^1^, Tingting Zhao^1^, Li Li^1^ & Pengfei Qi ^2^

^1^School of Civil Engineering and Architecture, Chuzhou University, Chuzhou, China, 239000.

^2^State Key Laboratory of Pharmaceutical Biotechnology, Nanjing University, Nanjing, China.

Running title: Temporal and spatial variations in the bacterial community composition in Lake Bosten.

----------------------------------

^*^ Corresponding author: Lei Zhang.

E-mail: leizhang2014@163.com; Tel. (+86) 550 3511822; Fax. 550 3511822.

Address: School of Civil Engineering and Architecture, Chuzhou University, 1 West Huifeng Road, Chuzhou, China, 239000.

**Supplementary materials**

**Fig. S1.** Environmental variables from twenty sediments in Lake Bosten

**^
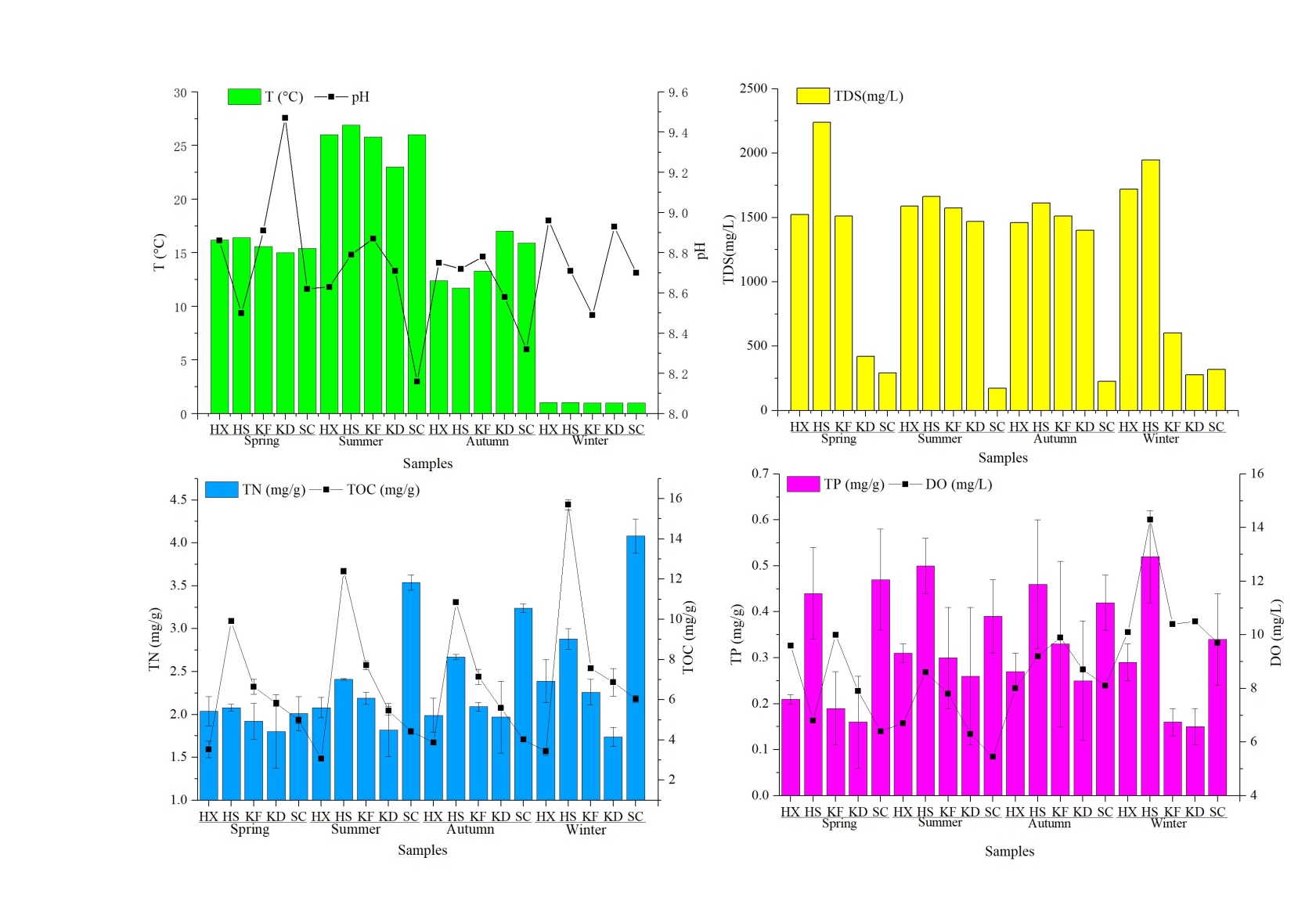
^**
